# Cytokinin signaling is involved in root hair elongation in response to phosphate starvation

**DOI:** 10.1080/15592324.2024.2305030

**Published:** 2024-01-24

**Authors:** Hirotomo Takatsuka, Toshiki Amari, Masaaki Umeda

**Affiliations:** a School of Biological Science and Technology, College of Science and Engineering, Kanazawa University, Kanazawa, Ishikawa, Japan; b Graduate School of Science and Technology, Nara Institute of Science and Technology, Takayama, Ikoma, Nara, Japan

**Keywords:** Root hair, phosphate starvation, cytokinin, response regulator, RSL4

## Abstract

Root hair, single-celled tubular structures originating from the epidermis, plays a vital role in the uptake of nutrients from the soil by increasing the root surface area. Therefore, optimizing root hair growth is crucial for plants to survive in fluctuating environments. Root hair length is determined by the action of various plant hormones, among which the roles of auxin and ethylene have been extensively studied. However, evidence for the involvement of cytokinins has remained elusive. We recently reported that the cytokinin-activated B-type response regulators, ARABIDOPSIS RESPONSE REGULATOR 1 (ARR1) and ARR12 directly upregulate the expression of *ROOT HAIR DEFECTIVE 6-LIKE 4* (*RSL4*), which encodes a key transcription factor that controls root hair elongation. However, depending on the nutrient availability, it is unknown whether the ARR1/12–RSL4 pathway controls root hair elongation. This study shows that phosphate deficiency induced the expression of *RSL4* and increased the root hair length through ARR1/12, though the transcript and protein levels of ARR1/12 did not change. These results indicate that cytokinins, together with other hormones, regulate root hair growth under phosphate starvation conditions.

## Results and discussion　

Root hairs are tubular extensions of epidermal cells, which facilitate the uptake of water and nutrients from the soil by increasing the root surface area.^1–3^ Thus, root hair elongation likely contributes to sustainable plant growth under fluctuating environments. Nutrient availability significantly affects root hair growth, and in general, insufficient levels of nutrients induce an increase in both the length and density of root hairs, thereby elevating the efficiency of nutrient uptake.^1–3^ Among the nutrients essential for plant growth, inorganic phosphate (Pi) significantly impacts root hair growth. In various plants, such as *Arabidopsis thaliana*, rice, tomato, rapeseed, spinach, and chickpea, Pi deficiency led to a dramatic increase in root hair length and density,^4–7^ implying that plants have developed a strategy to cope with Pi starvation by enhancing root hair growth.

Changes in the expression of *ROOT HAIR DEFECTIVE 6-LIKE 4* (*RSL4*), which encodes a basic helix-loop-helix (bHLH) transcription factor (TF), resulted in an increase or decrease in root hair length, highlighting the role of *RSL4* as a determinant for root hair elongation.^8^ It is also known that *RSL4* functions in promoting hair growth under nutrient-limiting conditions.^8^ Extensive studies have shown that multiple inputs converge at *RSL4* to regulate root hair elongation. These include 1) another bHLH TF, ROOT HAIR DEFECTIVE 6 (RHD6), which directly induces *RSL4*, thereby constituting the transcriptional pathway essential for root hair development^8^ 2) auxin, which enhances root hair elongation through the binding of the AUXIN RESPONSE transcription FACTORs, ARF5, 7, 8, and 19, to the promoter of *RSL4* ^9^; and 3) the ethylene-activated TFs, ETHYLENE INSENSITIVE 3 (EIN3), and its homolog ETHYLENE INSENSITIVE 3-LIKE 1 (EIL1), which also bind to the promoter of *RSL4* and upregulate its expression.^10^

**Figure 1.**
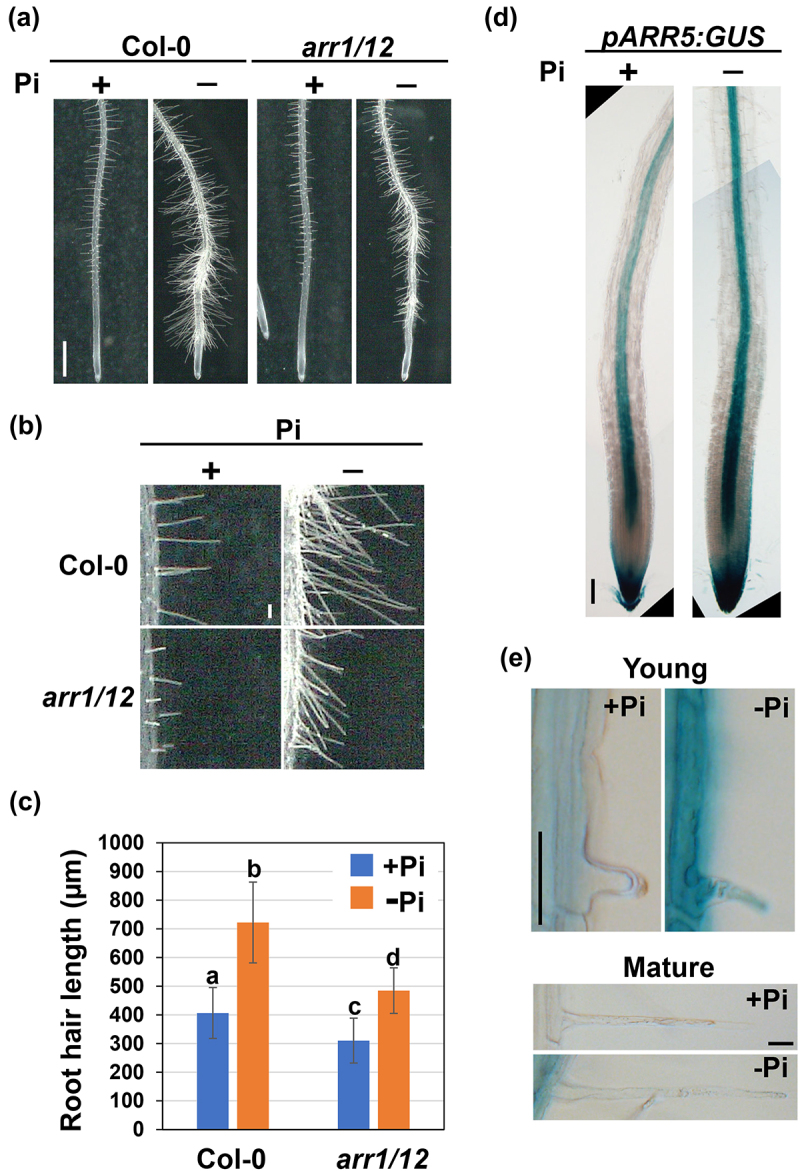
Pi starvation-induced root hair elongation is partially suppressed in *arr1/12*.

Recently, we identified the cytokinin (CK)-triggered signaling pathway that regulates *RSL4* expression and root hair elongation.^11^ The CK is perceived by the histidine kinase receptors ARABIDOPSIS HISTIDINE KINASEs (AHKs), and the signal eventually activates the B-type ARABIDOPSIS RESPONSE REGULATOR (ARR) TFs through a two-component system called “phosphorelay.”^12–17^ Among the 11 B-type ARRs, ARR1 played a major role in CK-induced root hair elongation by binding to the promoter of *RSL4* and enhancing its expression.^11^ The *rsl4* knockout mutant was insensitive to CK regarding root hair growth, as observed in the double knockout mutant of *ARR1* and its close homolog *ARR12*, hereafter referred to as *arr1/12* .^11^ Thus, it can be concluded that the ARR1/12–RSL4 pathway plays a pivotal role in promoting root hair elongation in response to CK.^11^ However, the evidence suggesting that it also serves as a module optimizing root hair elongation depending on the nutrient conditions remains elusive. The root hair growth of *arr1/12* on a Pi-deficient medium was observed to examine this possibility. The results revealed that the Pi deficiency-dependent increase in root hair length still occurred but to a lesser extent in *arr1/12* compared to the wild-type (Figure 1a–c), indicating the involvement of ARR1/12 in Pi starvation-triggered enhancement in root hair elongation. Then, to determine if Pi starvation activates CK signaling in root hair cells, we observed transgenic plants harboring the fusion gene of the 1.6-kb *ARR5* promoter and *GUS* reporter (*pARR5:GUS*), which is known to represent CK signaling.^18^ Expression of *pARR5:GUS* in the root tips was not affected by Pi deficiency, with the columella, lateral root cap, and vasculature exhibiting the highest GUS signals (Figure 1d). However, in young root hairs, Pi deficiency enhanced the intensity of the GUS signal, which was barely detectable under normal growth conditions (Figure 1e). In contrast, GUS signals were not detected in mature root hairs, regardless of Pi availability (Figure 1e). Taken together, these results suggest that Pi starvation activates CK signaling, possibly through upregulating B-type ARRs including ARR1/12, to promote root hair elongation.

(a) Roots of the wild-type (WT) (Col-0) and *arr1/12* seedlings. Five-day-old seedlings were transferred to the MS medium containing 0 mM (−Pi) or 1.25 mM (+Pi) KH_2_PO_4_ and grown for one more day. The scale bar represents 1 mm. (b) Magnified images of root hairs shown in a. The scale bar represents 100 µm. (c) Length of mature root hairs in the WT and *arr1/12*. Data are presented as mean ± SD (*n* > 150). The significance of the differences was determined using Tukey’s test (*P* < 0.05). Bars with various letters indicate significant differences from each other. (d) Root tips of GUS-stained transgenic plants harboring *pARR5:GUS*. Five-day-old seedlings were transferred to +Pi or −Pi medium and grown for one more day. The scale bar represents 100 µm. (e) Young and mature root hairs of GUS-stained transgenic plants harboring *pARR5:GUS* grown under the same conditions as d. The scale bar represents 100 µm.

**Figure 2.**
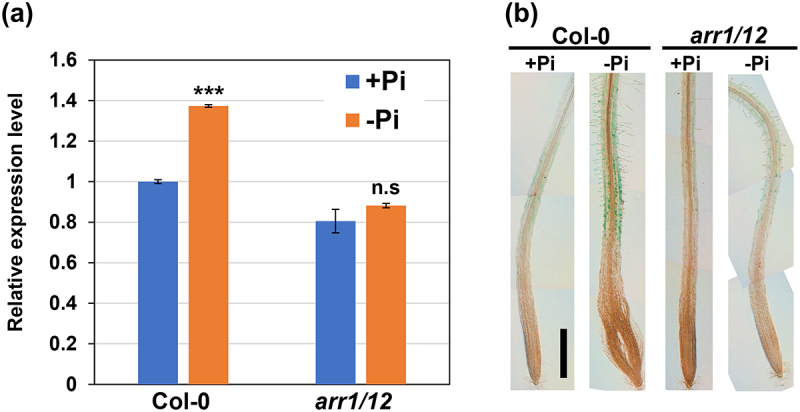
*RSL4* induction upon phosphate starvation is compromised in *arr1/12.*

A previous study demonstrated that the transcript levels of *RSL4* were elevated markedly in the absence of exogenous Pi,^8^ but the involvement of CK signaling was not studied. Therefore, qRT-PCR analysis was conducted using the RNA extracted from the roots of wild-type (WT) or *arr1/12* plants grown with or without the exogenous application of Pi. The data showed that Pi starvation significantly elevated the *RSL4* mRNA levels in the WT but not *arr1/12* (Figure 2a). The gene-trap GUS reporter line for *RSL4* ^8^ supported the above results. The GUS signals increased dramatically in the trichoblasts of the WT, but not markedly in *arr1/12* under Pi-deficient conditions (Figure 2b). These results suggest that Pi starvation activates ARR1 and ARR12, and consequently elevates the expression of *RSL4* to promote root hair growth.

(a) Transcript levels of *RSL4* quantified using qRT-PCR. Five-day-old seedlings of the wild-type (WT) (Col-0) and *arr1/12* were transferred to +Pi or −Pi medium and further for one more day. Total RNA was extracted from the root tips. Data are presented as mean ± SD (*n* = 3). The mRNA levels are indicated as relative values, with that for the WT seedlings grown on +Pi medium set to 1. Welch’s *t*-test was used to determine the significance of the differences between −Pi and +Pi for each genotype. ***P < 0.001; n.s.: not significant. (b) GUS staining of the roots of the WT and *arr1/12* seedlings harboring *pRSL4:GUS*. Five-day-old seedlings were transferred to +Pi or −Pi medium and grown for one more day. The scale bar represents 500 µm.

**Figure 3.**
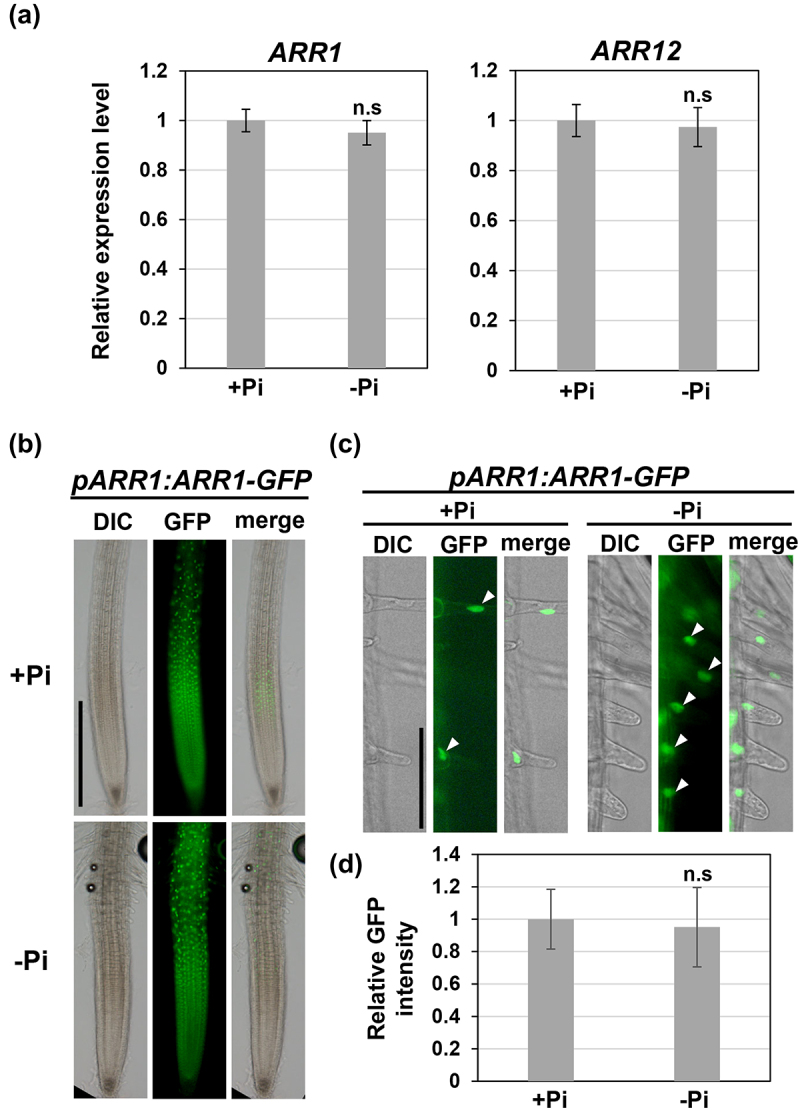
Pi deficiency does not alter the expression levels of ARR1 and ARR12.

To gain insight into the mechanisms by which Pi starvation activates the B-type ARRs, the mRNA and protein levels of ARR1 and 12 were determined under Pi-deficient conditions. qRT-PCR indicated that Pi starvation had no impact on their transcript levels in the roots (Figure 3a). Moreover, the GFP reporter line *pARR1:ARR1-GFP*, which expresses the ARR1-GFP fusion protein under the 2-kb *ARR1* promoter, displayed GFP fluorescence throughout the root tips regardless of Pi availability (Figure 3b). Under normal growth conditions, ARR1 accumulated in young root hairs, but disappeared in mature hairs.^11^ Therefore, the GFP fluorescence in the nuclei of the cells of the young hairs was quantified; the fluorescence intensity did not change when plants were grown under Pi-deficient conditions (Figure 3c, d). This and the above-mentioned data showed that CK signaling was enhanced without affecting mRNA or protein levels of ARR1/12 under Pi-limiting conditions, suggesting that Pi deficiency activates ARR1/12, but not increases their amounts, to upregulate CK signaling, thereby inducing *RSL4* and promoting root hair growth.

(a) Transcript levels of *ARR1* and *ARR12* were determined using qRT-PCR. Five-day-old wild-type seedlings were transferred to +Pi or −Pi medium and grown for one more day. Total RNA was extracted from the root tips. Data are presented as mean ± SD (*n* = 4). The mRNA levels are indicated as relative values, with that for the roots grown on +Pi medium set to 1. Welch’s *t*-test was used to determine the significance of the differences with and without Pi; n.s.: not significant. (b) Images of root tips harboring *pARR1:ARR1-GFP*. Five-day-old seedlings were grown on +Pi or −Pi medium for 1 day. The scale bar represents 500 µm. (c) Magnified images of elongating root hairs shown in b. White arrowheads indicate the nuclei in the cells of the elongating root hair. The scale bar represents 50 µm. (d) The intensity of the GFP fluorescence in elongating hair cells carrying *pARR1:ARR1-GFP*. Data are presented as mean ± SD (*n* = 20). The intensities of the GFP fluorescence were indicated as relative values, with that for the roots grown on +Pi medium set to 1. Welch’s *t*-test was used to determine the significance of the differences between −Pi and +Pi; n.s.: not significant.

Notably, the *arr1/12* mutant responded to Pi starvation regarding root hair growth, albeit to a lesser extent than the WT (Figure 1a–c). The involvement of other B-type ARRs can explain this result. Indeed, it was recently found that ARR2 has a non-negligible role in CK-induced hair elongation.^11^ Another possibility is that CK-independent signaling(s) also work in the Pi starvation response. Auxin and ethylene function in increasing root hair length under low Pi conditions,^19,20^ suggesting that these hormones are also associated with Pi deficiency-dependent root hair growth. In *arr1/12*, Pi starvation did not induce *RSL4* but promoted root hair elongation (Figures 1c, 2a), suggesting that factor(s) other than *RSL4* are also involved in Pi starvation response independently of ARR1/12, possibly through enhancing auxin and/or ethylene signaling to promote root hair growth. Favoring this notion, root hair elongation was subtly yet significantly enhanced in the *rsl4* loss-of-function mutant subjected to low Pi conditions.^20^ The mechanism by which multiple hormonal pathways coordinate root hair growth depending on exogenous Pi level is an interesting question for future studies.

The process by which Pi starvation activates CK signaling remains unknown. Given that the protein levels of ARR1 and ARR12 were not altered irrespective of Pi availability (Figure 3b–d), one possibility is that CK levels increase in response to Pi deficiency. Although previous studies demonstrated that CK levels rather decreased in whole roots under low Pi conditions,^21,22^ it is conceivable that CK biosynthesis and/or transport are locally enhanced to increase CK amounts in elongating root hairs. Another possibility is that other factors controlling the phosphor-relay cascade respond to Pi starvation, activating CK signaling. To test these possibilities, the quantification of CKs, as well as the mRNA and protein levels of CK biosynthesis-related enzymes, CK receptors (AHKs), His phosphor-transfer proteins (AHPs), and A-type ARRs can unravel the whole picture of CK-dependent pathway regulating root hair elongation under Pi-deficient conditions. Clarifying how nutrient availability affects hormonal biosynthesis, transport, and signaling in distinct cell types will pave the way for a better understanding of plant strategies to sustain optimal growth and development in a challenging environment.
